# Live weight, conformation, carcass traits and economic values of ram lambs of Red Maasai and Dorper sheep and their crosses

**DOI:** 10.1007/s11250-016-1168-5

**Published:** 2016-10-14

**Authors:** E. Zonabend König, J. M. K. Ojango, J. Audho, T. Mirkena, E. Strandberg, A.M. Okeyo, J. Philipsson

**Affiliations:** 1 Department of Animal Breeding and Genetics, Swedish University of Agricultural Sciences, P.O. Box 7023, 750 07 Uppsala, Sweden; 2 International Livestock Research Institute, Nairobi, Kenya; 3 School of Animal and Range Sciences, College of Agriculture, Hawassa University, Hawassa, Ethiopia

**Keywords:** Sheep breeds, Evaluation, Lamb growth, Assessments, Carcass merits, Heterosis

## Abstract

Meat production is the most important trait in the breeding objectives of sheep production in East Africa. The aim of this study was to investigate breed differences in live weight, conformation, carcass traits and economic values for meat production among Red Maasai and Dorper sheep and their crosses. In total, 88 ram lambs, which were reared at the ILRI experimental station, Kapiti plains Estate in Central Kenya, were used for the study. The lambs were slaughtered at Kenya Meat Commission (KMC) at about 1 year of age. Prior to slaughter, the lambs were weighed, measured and assessed by experienced evaluators, and at the abattoir carcass traits were recorded. Large breed differences were found for most traits. Dorper lambs were heavier at delivery for slaughter and had better carcass grade but lower dressing percentage and fat levels than Red Maasai. Crossbreds were generally better than the parental breeds. Evaluators were willing to pay more for the Dorper lambs for slaughter although carcass weights later were shown not to be higher than for Red Maasai. Evaluators undervalued Red Maasai lambs by 8–13 % compared to Dorper lambs according to the prices quoted per kilogramme live or carcass weight by KMC. Live weight was better than any other live measure in predicting carcass weight. Due to the overall higher ranking of the crossbred lambs for meat production, Dorper may be useful as a terminal sire breed for crossing with Red Maasai ewes.

## Introduction

Small ruminant meat plays an important role in many rural African households, especially in pastoral systems (Juma et al. 2010). In a recent UN-led study on “African Livestock Futures”, small ruminant meat production in pastoral systems was shown in different scenarios to have the potential of increasing production by five to six times by 2050 relative to the production levels of year 2000 (Herrero et al. 2014). The relative impact of various small ruminant breeds on the projected increased productivity may however differ.

One popular small ruminant breed raised in the semi-arid and arid lands of Kenya is the Red Maasai sheep, an East African fat-tailed sheep breed. Red Maasai sheep are, however, declining among their traditional keepers, due to widespread crossbreeding with the Dorper breed, initially imported from South Africa for improvement of meat production. Both Red Maasai and Dorper sheep and their crosses are important to the farmers for different reasons (Zonabend König et al. 2016). Red Maasai sheep are appreciated by their keepers for their ability to survive and produce under harsh environments, and the Dorper is valued for its higher growth rate potential and larger mature size, whereas the crosses have good survival rates compared to Dorper and relatively better growth rates than the Red Maasai (Baker et al. 2002). Comparative studies of Red Maasai and Dorper sheep indicate that in harsh or humid environments, differences in performance between the breeds tend to be smaller in magnitude (Baker et al. 2002; Mugambi et al. 2005; Zonabend König et al. 2016).

A large body size as an indicator of good growth rate is an important trait when marketing sheep within different communities in the arid lands of Eastern Africa (Gizaw et al. 2010; Mtimet et al. 2014). Mtimet et al. (2014) showed that in the absence of live weight data, breed and visually assessed size are the most important factors in determining the price for live animals. It is important for farmers to know what the market (middlemen) prefer in order to align their breeding strategy with the market demands (Lambe et al. 2008; Katiku et al. 2013; Ojango et al. 2014).

Comparative studies on carcass traits of indigenous sheep breeds and their crosses with improved breeds within Eastern Africa are rare. Results from carcass studies on indigenous sheep breeds in Ethiopia show some breed differences for both carcass weight and fat deposition (Ermias et al. 2002; Ermias et al. 2006). Crossbred Blackhead Ogaden with Dorper has been reported to have improved carcass weights by 15 % compared to pure Blackhead Ogaden (Tsegay et al. 2013). There are, however, no comparative studies on carcass traits of Red Maasai and Dorper sheep and their crosses.

With the growing importance and significance of the sheep industry in Eastern Africa (FAO 2016), and the use of indigenous breeds and their crosses in pastoral systems, the aims of this study were to (i) investigate potential differences in live weight and carcass traits among ram lambs of the Red Maasai and Dorper breeds and their crosses; (ii) assess the economic values of ram lambs for slaughter for the same breed groups, and (iii) provide information on the accuracy in prediction of carcass traits from live weight and conformation traits, objectively measured or subjectively assessed, prior to delivery for slaughter.

## Material and methods

### Experimental animals and recording of traits

Ram lambs of Red Maasai and Dorper breeds and their crosses were raised at the International Livestock Research Institute ranch at Kapiti Plains Estate (Kapiti), Kenya, and slaughtered at the largest abattoir in the country, the Kenya Meat Commission (KMC) on the outskirts of Nairobi.

Animals at Kapiti are reared on natural grasslands in a semi-arid environment with limited supplementation by feeding hay to nursing ewes and their young lambs during periods of extreme droughts. The sheep are maintained as a nucleus flock for selection and improvement of sheep under arid conditions as outlined by (ILRI 2016). All animals are routinely provided with basic vaccinations and are washed in dips with acaricides once a week against ecto-parasites, especially ticks. Animals observed to be suffering from severe haemonchosis were treated with anthelmintics. Lambing is planned to occur twice in a year over a 6-week period in the seasons when pastures are more available. Lambs are weaned in batches at about 3 months of age, with variation in age of the lambs resulting from differences in the day of lambing. The tails of all lambs were docked before the age of 2 months.

Ram lambs used in this study were from two batches: the first batch (batch 1) comprised 27 lambs born in August–September 2011 and the second batch (batch 2) included 61 lambs born in November–December the same year. They comprised 18 (7 and 11 in batches 1 and 2) purebred Red Maasai lambs (RRRR), 24 (3 and 21) lambs of 50 %-crosses (DDRR), 32 (11 and 21) lambs of 75 % Dorper (DDDR) and 14 (6 and 8) purebred Dorper (DDDD) lambs. All breed groups were thus represented in both batches. The DDRR lambs included 13 F1 animals comprising both reciprocal crosses (Dorper males × Red Maasai ewes and Red Maasai males × Dorper ewes) and 11 F2 animals. The backcrosses (DDDR) were included as they constituted a large portion of the crossbreds and would support any effects of crossbreds and are also on high demand by pastoralists in the surrounding area.

Recorded traits are presented in Table 1. Birth weight (BW) was recorded in all lambs (used to compare with later body measures). Prior to delivery for slaughter at the KMC, animals were weighed with a scale and measured using a standard tape to determine their live weight (LW), heart girth (HG) and body length (BL). At the same time, the ram lambs were subjectively assessed by experienced evaluators to determine their measures of weight (LWA), conformation (CA), body score (BSA) and total score (TSA). The first batch was assessed by one butcher and the second batch was assessed by the same butcher, a middleman (trader) and a farmer to allow views of different professions. In addition, the evaluators were asked to quote the prices that they would be willing to pay for each of the lambs if it were to be used for slaughter. The value was quoted in Kenya Shilling (KES) and was considered as the market value of the lamb at the time of slaughter (WPA). It includes primarily the expected carcass value, but may also include the value of offals, hide and skin. Live weight measured on delivery day to the abattoir was referred to as delivery weight (DW). A protocol was developed to be used in the evaluation and grading of the sheep carcasses that was carried out by trained livestock evaluators at the KMC. Dressed carcasses of animals were weighed to obtain a hot carcass weight and then chilled and the cold carcass weight (CWT) was recorded the day after slaughter. Cold carcasses were assessed for their fat level (CFL), muscle formation (CMF) and grade (CG). Routinely, the KMC does not grade carcasses of small ruminants and pays a fixed price of KES 150 (USD 1.79) per kilogramme of DW. Alternatively, KMC offers a flat rate of KES 315 (USD 3.75) per kilogramme hot carcass weight for the small ruminants.

**Table 1:** Recorded traits with units and abbreviations (Abbr) presented per batch and with average age (in days) of ram lambs at recordings

Trait	Units	Abbr.	Batch	Birth	Evaluation	Delivery	Slaughter
			1	Aug–Sept	317	317	322
			2	Nov–Dec	369	380	384
Birth weight	(kg)	BW		X			
Live weight	(kg)	LW			X		
Heart girth	(cm)	HG			X		
Body length	(cm)	BL			X		
Willingness to pay	(KES)^a^	WPA			X		
Live weight assessment	(1–5)	LWA			X		
Conformation assessment	(1–5)	CA			X		
Body score assessment	(1–5)	BSA			X		
Total score assessment	(1–5)	TSA			X		
Delivery live weight	(kg)	DW				X	
Growth rate to delivery	(g/day)^b^	GR				X	
Cold carcass weight	(kg)	CWT					X
Carcass fat level	(1–3)	CFL					X
Carcass muscle formation	(1–3)	CMF					X
Carcass grade	(1–7)^c^	CG					X
Dressing percentage	(%)^d^	DP					X

^a^1 USD = 84 KES for both batches

^b^GR = (DW − BW)/age × 1000

^c^Lower value describes a preferred carcass grade

^d^DP = CWT/DW × 100

Average age at slaughter was 364.8 days (359.6 for RRRR, 376.2 for DDRR, 363.8 for DDDR and 354.6 days for DDDD).

### Statistical analysis

All analyses were performed using the software “The R Project for Statistical Computing” (R Core Team 2015). Because the quantile-quantile plots and histograms of residuals for all traits were normally or close to normally distributed, linear models were used. The R packages Companion to Applied Regression (Fox and Weisberg 2011) and the linear and nonlinear mixed effects models (Pinheiro et al. 2015) were used for the general linear and mixed model analyses to adjust for fixed effects, and the package lsmeans (Lenth 2013) was used for estimation of the least squares means to account for unequal size of subclasses. The following three linear models were used for analyses of factors that potentially would influence live animal and carcass traits:

1 $$ {y}_{ijkl}=\mu +{\mathrm{Breed}}_i+{\mathrm{Batch}}_j+{b}_{1i}{\mathrm{Age}}_k\left({\mathrm{Breed}}_i\right)+{e}_{ijkl} $$

2 $$ {y}_{ijklm}=\mu +{\mathrm{Breed}}_i+{\mathrm{Batch}}_j+{b}_{1i}{\mathrm{Age}}_k\left({\mathrm{Breed}}_i\right)+{b}_{2l}{\mathrm{LiveMeasure}}_l+{e}_{ijklm} $$

3 $$ {y}_{ijklmn}=\mu +{\mathrm{Breed}}_i+{\mathrm{Batch}}_j+{b}_{1i}{\mathrm{Age}}_k\left({\mathrm{Breed}}_i\right)+{\mathrm{Evaluator}}_l+{\mathrm{ID}}_m+{e}_{ijklmn} $$

where *y*_*ijkl*_ in (1) is the trait of interest: BW, LW, HG, LBL, WPA, DW, GR, CWT, CFL, CMF, CG or DP; in (2), *y*_*ijklm*_ is the carcass trait of interest: GR, CWT, CMF, CFL, CG, DP or WPA (mainly reflecting carcass value); and in (3), *y*_*ijklmn*_ is the subjectively assessed traits: WPA, LWA, CA, BSA or TSA.

In each model, *μ* is the overall mean for the trait; *Breed*_*i*_ is the effect of the *i*th breed group (*i* = RRRR, DDRR, DDDR, DDDD); *Batch*_*j*_ is the effect of *j*th batch (*j* = batches 1 and 2); *b*_*1i*_ is the regression of the trait on age (in days at recording) nested within breed *i* (*Age*_*k*_(*Breed*_*i*_)); *b*_*2l*_ is the regression of the trait on *LiveMeasure*_*l*_ (*l* = an objective measure LW, HG, BL or DW or subjective measure WPA, LWA, CA, BSA or TSA); *Evaluator*_*l*_ is the fixed effect of the *l*th evaluator (*l* = 1–3); and *ID*_*m*_ as the random effect of lamb, where ID∼ND(0, *σ* ^2^_ID_); *e* is the random residual error∼ND (0, *σ* ^2^_e_). Adjustment of each trait for age is done to the overall mean age of all the animals at the time of recording the specific trait. For the models (1) and (2), adjusted coefficients of determination (adjusted *R* ^2^) were calculated according to (Fox and Weisberg 2011) based on Wherry (1931), as the data set was rather small.

Model (1) was used to estimate differences between breed groups. Based on results from (1), an estimate of heterosis was calculated for the live animal and carcass traits by first dividing the least squares mean of DDRR by the average least squares means for RRRR and DDDD, and given as percent of that average. The heterosis was solved for the expectation of this value: (propF_1_ × heterosis + propF_2_ × heterosis × 0.5), where propF_1_ was 13/24 and propF_2_ was 11/24. Associations between traits were analysed by using Pearson correlations of residuals from (1). Model (2) was employed to study how objective live measures of weight and conformation or subjective assessments can explain the variation in carcass traits including assessed economic value of the lamb (WPA). For the assessed traits, records based on the common evaluator between batches 1 and 2 were used. Model (3) was used to study the effect of evaluator on the assessed traits.

## Results

### Significance of model factors

Results from (1) showed that effects of breed groups and age were significant (*p* < 0.05) or close to significant (*p* < 0.1) for almost all traits (not shown in tables), whereas effects of batch were small despite 2 months difference in age at slaughter. Live animal measures (LW, HG, BL and DW) were shown in (2) to significantly predict GR, CWT, CMF and WPA. Model (3) showed that evaluators significantly *(p* < 0.001) affected all subjectively assessed traits including WPA except for CA.

### Breed group differences

Results in Table 2 show that pure Red Maasai had a 0.7 kg lower birth weight and a 2.1 kg lower live weight (LW) (*p* < 0.08) and weight at delivery (DW) (*p* < 0.07) than pure Dorper. Both crossbred groups exceeded the pure breeds in LW and DW. As regards conformation, Red Maasai had significantly shorter body length (BL) than the other breed groups, whereas heart girth (HG) showed only small differences between the pure breeds. Dorper and the crosses were offered significantly higher prices by the evaluators (WPA), 12 % or KES 519 (US$ 6.18) more than for pure Red Maasai lambs. The latter also produced carcasses with significantly lower scores for carcass grade (CG) compared with Dorper (Table 3). However, carcass weight (CWT) did not differ between the pure breeds, whereas the 50 %-crosses (DDRR) were significantly heavier, 12–13 %, than the pure breeds. The dressing percentage (DP) was significantly higher for Red Maasai (41.6 %) compared with Dorper (39.1 %), whereas the crosses did not significantly differ from any of the parental breeds.

**Table 2:** Least squares means ± standard error and approximate heterosis (%) for live animal measures by level of breeds based on model (1) for all traits except for WPA that is based on model (3)

Breed	BW	LW	HG	BL	WPA	DW
RRRR	3.40 ± 0.12^a^	36.07 ± 0.97^a^	79.61 ± 0.88^ab^	57.85 ± 0.87^a^	4321 ± 200^a^	36.35 ± 1.02^a^
DDRR	3.79 ± 0.12^b^	39.47 ± 0.97^b^	80.99 ± 0.89^b^	62.36 ± 0.87^b^	4844 ± 193^b^	39.89 ± 1.02^b^
DDDR	4.05 ± 0.09^b^	38.59 ± 0.77^b^	79.61 ± 0.70^ab^	61.83 ± 0.68^b^	4844 ± 171^b^	38.93 ± 0.83^b^
DDDD	4.11 ± 0.14^b^	38.16 ± 1.13^ab^	78.25 ± 1.03^a^	60.98 ± 1.01^b^	4840 ± 213^b^	38.48 ± 1.18^ab^
Heterosis	1.2	8.2	3.4	6.5	7.5	8.6

*BW* birth weight, *LW* live weight, *HG* heart girth, *BL* body length, *WPA* willingness to pay, *DW* delivery live weight

^a,b^Means within column with different superscripts are significantly different at *p* < 0.05

**Table 3:** Least squares means ± standard error and approximate heterosis (%) for growth rate and carcass traits by level of breeds based on model (1)

Breed	GR	CWT	CFL	CMF	CG^c^	DP
RRRR	92.00 ± 2.74^a^	15.11 ± 0.49^a^	2.12 ± 0.11^b^	2.02 ± 0.18^a^	5.03 ± 0.33^b^	41.61 ± 1.10^b^
DDRR	101.31 ± 2.73^b^	16.50 ± 0.49^b^	2.04 ± 0.11^ab^	2.56 ± 0.18^b^	4.74 ± 0.33^ab^	41.35 ± 1.10^ab^
DDDR	97.53 ± 2.22^b^	15.80 ± 0.40^ab^	1.94 ± 0.09^ab^	2.17 ± 0.14^a^	4.24 ± 0.27^a^	40.66 ± 0.89^ab^
DDDD	96.19 ± 3.15^ab^	15.03 ± 0.57^a^	1.81 ± 0.13^a^	2.25 ± 0.20^ab^	3.97 ± 0.38^a^	39.09 ± 1.27^a^
Heterosis	10.0	12.3	4.9	25.8	6.9	3.2

*GR* growth rate to delivery, *CWT* cold carcass weight, *CFL* carcass fat level, *CMF* carcass muscle formation, *CG* carcass grade, *DP* dressing percentage

^a,b^Means within column with different superscripts are significantly different at *p* < 0.05

^c^Lower value of CG meaning better carcass grade

Based on results from the 50 %-crosses (DDRR), positive heterosis effects were realized for all traits, and on an average, the 50 %-crosses were 8–9 % better than the mean of the pure breeds in all traits.

### Influence of live animal traits on carcass traits and willingness to pay

Table 4 shows the amount of variation in growth rate, carcass traits and WPA that was explained by different factor combinations (breed, batch, age, live measures and assessments). LW was the live measurement explaining most of the variation in carcass traits and WPA, accounting for 41–49 % of the variation in CWT and GR, 14 % in CMF and 18 % in WPA. When LW was included in the model, HG, BL or DW did not have an effect on the carcass traits (results not shown). However, LW did not explain any of the variation in DP, CG or CFL. When breed group effects were adjusted for, LW still explained most of the variation, between 12 and 44 % in GR, CWT and WPA. Overall, breed groups accounted for 6–12 % of the observed variation in growth, carcass traits and WPA.

**Table 4:** Adjusted coefficients of determination for growth and carcass traits and for willingness to pay using different factor combinations based on model (2)

Factor combination	GR	CWT	CG	CFL	CMF	DP	WPA
Batch + Age	0.457	0.020	0.251	0.099	0.121	0.000	0.343
Batch + Age + LW	0.951	0.435	0.252	0.111	0.259	0.000	0.522
Batch + Age + LWA	0.477	0.025	0.252	0.092	0.116	0.000	0.459
Batch + Age + WPA	0.606	0.103	0.263	0.101	0.178	0.000	
Breed + Batch + Age(Breed)	0.518	0.120	0.311	0.185	0.199	0.057	0.460
Breed + Batch + Age(Breed) + LW	0.953	0.478	0.308	0.216	0.271	0.067	0.576
Breed + Batch + Age(Breed) + LWA	0.536	0.126	0.305	0.187	0.191	0.049	0.540
Breed + Batch + Age(Breed) + WPA	0.633	0.191	0.305	0.174	0.224	0.050	

*LW* live weight, *WPA* willingness to pay, *LWA* live weight assessment, *GR* growth rate to delivery, *CWT* cold carcass weight, *CG* carcass grade, *CFL* carcass fat level, *CMF* carcass muscle formation, *DP* dressing percentage

Although much less informative than LW, WPA was the most informative attribute of the variation in carcass traits, 7–8 % of the variation in CWT when data were adjusted for breed, batch and age, but much less informative of the other carcass traits. Subjective assessments of live weight (LWA) and other conformation traits (CA, BSA and TSA) explained no more than 2 % of the variation in growth and carcass traits when breed was adjusted for.

### Residual correlations between weights, conformation and growth traits

Weights, conformation and growth traits are closely related (Table 5). The residual correlation between LW and CWT was 0.64. It was 0.69 between LW and HG, whereas it was only 0.31 between LW and BL. The correlations between CWT and the two conformation measures were 0.47 (HG) and 0.24 (BL). All correlations between LW and live conformation measures with CG and DP were weak and non-significant.

**Table 5:** Residual correlations between live weight, conformation, growth rate, carcass traits and willingness to pay based on model (1) and the common evaluator between batches 1 and 2 in the upper right triangle

	BW	LW	HG	BL	WPA	DW	GR	CWT	CFL	CMF	CG	DP
BW		0.49	0.40	0.31	0.05	0.44	0.30	0.29	0.07	0.28	−0.26	−0.12
LW	***		0.69	0.31	0.47	0.98	0.95	0.64	0.22	0.32	−0.09	−0.20
HG	***	***		0.27	0.33	0.66	0.64	0.47	0.08	0.15	−0.09	−0.12
BL	**	**	*		0.07	0.29	0.26	0.24	0.09	0.19	−0.16	−0.02
WPA	ns	***	**	ns		0.48	0.50	0.31	0.00	0.21	−0.06	0.04
DW	***	***	***	**	***		0.98	0.61	0.16	0.29	−0.07	−0.17
GR	**	***	***	*	***	***		0.58	0.18	0.26	−0.02	−0.15
CWT	**	***	***	*	**	***	***		0.23	0.42	−0.11	0.32
CFL	ns	*	ns	ns	ns	ns	ns	*		0.09	−0.02	−0.04
CMF	**	**	ns	ns	*	**	*	***	ns		−0.31	0.14
CG	*	ns	ns	ns	ns	ns	ns	ns	ns	**		0.02
DP	ns	ns	ns	ns	ns	ns	ns	**	ns	ns	ns	

Significance levels are shown in the lower left triangle

*BW* birth weight, *LW* live weight, *HG* heart girth, *BL* body length, *DW* delivery live weight, *GR* growth rate to delivery, *CWT* cold carcass weight, *DP* dressing percentage, *CFL* carcass fat level, *CMF* carcass muscle formation, *CG* carcass grade, *WPA* willingness to pay

Significance levels: ***p < 0.001, ** *p* < 0.01, *p < 0.05, ns = nonsignificant

## Discussion

### Breed differences in live weight and carcass traits

The 2 kg, or 6 %, higher live weight of Dorper lambs at about 1 year of age compared to Red Maasai confirms the superiority of Dorper live weights found in previous studies (Kiriro 1994; Baker 1998; Baker et al. 2002; Mugambi et al. 2005; Zonabend König et al. 2016). However, Zonabend König et al. (2016) indicated even larger differences between the two breeds, especially in semi-arid environments, which also characterize the environment of this study. A reason for the smaller difference in live weight between the two breeds in the current study may be that the Kapiti herd of Red Maasai sheep has undergone a significant improvement through selection for live weight and conformation in the past decades (A.M. Okeyo, personal communication, 2015).

Despite the difference in live weight, no significant difference was observed in carcass weight between the two pure breeds in the present study, 15.1 vs 15.0 kg cold carcass weight for Red Maasai and Dorper. This result has been obtained due to a higher (*p* < 0.05) dressing percentage of Red Maasai, 41.6 vs. 39.1 % for Dorper. Significantly leaner (CFL) carcasses of Dorper may have contributed to the lower dressing percentage. As a fat-tailed sheep breed, the Red Maasai has fat deposits around the tail and rump contributing to a higher dressing percentage, whereas the fat in Dorper carcasses are more distributed around the internal organs and are easier to separate at slaughter. It may also be speculated whether larger and heavier head, offals and skin of the Dorper lambs contribute to the lower dressing percentage of this breed. Unfortunately, there are no previous studies appropriate to compare with from the region except the Ethiopian study on crosses between Dorper and Blackhead Ogaden, where the Dorper crosses clearly had the highest dressing percentage (Tsegay et al. 2013).

The higher fat levels and, thus, also higher dressing percentage of Red Maasai may be an expression of its well-known adaptation characteristics necessary to withstand harsh climates with intermittent droughts and for reproduction ensuring the animal has energy depots for use when needed.

Carcass grades in the present study were significantly better (*p* < 0.01) for Dorper than for Red Maasai. However, the standards of grading were less rigorous than the case under international standards, and the differences may therefore be treated more lightly. The scores for muscle formation were also slightly higher for Dorper. As this study, which comprised a limited number of purebred lambs, is to the best of our knowledge the first one reported regarding comparison of carcass traits of Red Maasai and Dorper sheep, and since carcass weights unexpectedly did not differ between the breeds, further studies on slaughter lambs of Red Maasai and Dorper lambs are warranted.

### Crossbreeding effects

The crosses were superior to the two parent breeds for most traits. Approximate heterosis effects based on the 50 %-crosses (DDRR) were estimated at 6.5 % for live traits and about 12 % for CWT. These results can be compared with those by Kiriro (1994), where Red Maasai and Dorper crossbred lambs were 4.5 % heavier at birth and 11 % at weaning than the average of the parental breeds. Results of the backcrosses (DDDR) are generally intermediate between pure Dorper and the 50 % crosses. For dressing percentage, effects of heterosis seem marginal. Both crossbred groups have results regarding dressing percentage more similar to Red Maasai than to Dorper. The superior effects of the crosses indicate one reason why crossbreeding between Red Maasai and Dorper has become very common in the study area. A production system where the more resilient and low maintenance Red Maasai ewes are used as dams to faster growing crosses with Dorper may be ideal.

### Economic evaluations

The economic values of the lambs, as expressed by the willingness to pay assessment by three experienced evaluators, one farmer, one middleman and one butcher, showed a significant (*p* < 0.01) difference in favour of Dorper over Red Maasai by 12 % despite that no difference was detected later in carcass weight. This confirms earlier studies of Mtimet et al. (2014) and of Zonabend König et al. (2016), where Dorper sheep were better priced than Red Maasai at farm gate and in local livestock markets. In the latter study of the value of potential breeding ewes, Dorpers were assigned a 9 % higher value in the least market-oriented area, whereas the more commercial farmers were willing to pay almost twice as much for a Dorper ewe as for a Red Maasai. The WPA value may in this case, however, include much more than just carcass value, such as expected breeding potential including appearance of the animal, skin and wool quality.

Crossbreds were given the same WPA value as pure Dorper, about KES 4840 (USD 57.62) in this study. However, in the study of breeding ewes by Zonabend König et al. (2016), the crossbreds were valued at an intermediate price, a little closer to Red Maasai than the price of Dorper. Also, Kosgey et al. (2008) reported that exotic and crossbred sheep fetched higher prices than indigenous animals. The price quoted by KMC was a flat rate of 150 KES per kilogramme live weight or 315 KES per kilogramme hot carcass weight, which corresponds to approximately 325 KES (USD 3.87) per kilogramme cold carcass weight. If these figures are applied on the present data, evaluators were willing to pay the lambs approximately 900–1100 KES (USD 10.72–13.10), or 19–26 %, less than the quoted price per kilogramme live weight by KMC. If comparisons were made with quoted prices per kilogramme carcass weight, without any premium for better carcass grades, the Red Maasai lambs were undervalued by 545 KES (USD 6.49), or 13 %, compared to the Dorper lambs. The price difference between the two quoted systems of KMC amounted to a 540–880 KES (USD 6.43–9.52), or 10–15 %, higher payment according to the live weight quotes, again the highest difference was for Dorper. These differences should cover costs that normally farmers, middlemen or butchers take as they operate at primary or secondary markets compared to the commercial abattoirs. However, Red Maasai lambs seem again to be undervalued compared to Dorper lambs, in this case by 340 KES (USD 4.05), or 8 %. Crossbred lambs were also undervalued compared to pure Dorper, but less so than Red Maasai.

Thus, large differences exist in how evaluators value the breeds, and where Red Maasai lambs were most undervalued, compared to payments according to empirical weights. Large differences were also shown among the assessors’ willingness to pay for the same animals. In the present study, the number of evaluators differed between the two batches, but the participating butcher was common to both batches. Thus, the effects of evaluator on the results were minimized by the statistical model used. Due to the apparent differences among evaluators (middlemen), it is important that farmers strive for delivery of lambs directly for slaughter and that payment is based on objective measures of weight. Middlemen often control the market and exploit the farmers by buying sheep at very low prices (Kosgey et al. 2006). Development of farmers’ associations would also be beneficial for farmers in order to better inform on the real market prices of the animals.

Carcass grades were better for Dorper and the crosses, and these breed groups would be favoured if grades were considered in the payment scheme. This may be more so given that exports and local high-end markets prefer carcasses of younger animals with better grades. The markets may, however, vary as regards preferences. The leaner carcasses of Dorper may attract customers close to cities, whereas Red Maasai carcasses may be more suited for rural local markets (Katiku et al. 2013). A research report about exports and market structure for live animals in Ethiopia showed that small traders and export abattoirs value lambs for slaughter differently. Besides weight, small traders valued skin condition highly, which was not as relevant for export abattoirs (Hailemariam et al. 2009).

### Live animal measures for prediction of carcass weight

Weighing animals intended for slaughter is the best way to predict carcass weight. Live weight (LW) explained 36 % of the variation in CWT when adjusting also for breed (Table 4) and the residual correlation between LW and CWT was 0.64 (Table 5). If a weighing scale is not available, a conformation measure may be used as a proxy. Heart girth (HG) showed the highest residual correlation with CWT, 0.47, among the conformation measures taken. Subjective assessments of weight hardly explained any variation in CWT. Willingness to pay (WPA) was the best subjective predictor, explaining 7 % if breed, age and batch were adjusted for. Large differences between evaluators were, however, observed for all live assessment traits except for conformation assessment (CA).

As regards correlations with LW among the live measures, again HG showed the highest residual correlation, 0.69, which is adjusted for the fixed factors breed, age and batch. A study on Dorper ram lambs by Fourie et al. (2002) indicated higher values, with a direct correlation between heart girth and live body weight of 0.8. However, it was based on data with high standard errors of the means of studied variables.

A weighing scale would, according to our study, be highly preferred when deciding which lambs should be sent to slaughter. It also helps the farmers to get lambs more correctly priced when selling their lambs live to middlemen or butchers. A tape for measuring heart girth would be a substitute for a scale, but with lower expected accuracy.

### Aspects on a breeding programme

All ram lambs in this study were born and raised at the Kapiti Plains Estate Limited (Kapiti), which is characterized by semi-arid climate. Larger differences in live weight between Red Maasai and Dorper would therefore have been expected following results of previous studies in the same environment (Baker 1998; Baker et al. 2002). However, the earlier studies reflect the population characteristics as they were about two decades ago and much may have changed since then. Kapiti mainly sources rams from within its own flocks, but also from other farms in the country. Excess animals not used for replacement at Kapiti are normally sold to local farmers interested to obtain breeding animals from the ranch. Thus, the genetic material from Kapiti is widespread in the country. Ram lambs in this study may, therefore, be considered representative for lambs raised in the region.

Several important results relating to the design of a breeding programme were obtained from the current study. Firstly, the advantages of Dorper as regards production of crossbred lambs for slaughter have been clearly demonstrated. Thus, the use of Dorper as a terminal sire breed to be crossed with Red Maasai ewes might be beneficial. Secondly, pure Red Maasai lambs seem to be undervalued by live assessments, 340–545 KES (USD 4.05–6.49) compared with Dorper lambs given that payment is only per kilogramme of empirical live or carcass weight. These discrepancies in economic values need to be considered when evaluating Red Maasai vs. Dorper and their crosses for slaughter and in the choice of a breeding strategy. Recording schemes to support both management decisions by the farmers, when selecting lambs for slaughter, as well as providing live weight data for selection purposes, should emphasize the use of as objective weight information as possible.

## Conclusions

Large differences in live weight prior to slaughter were found with higher weights of Dorper over pure Red Maasai. Crosses were better than both parental breeds for most traits. Carcasses of Dorper lambs were not heavier than those of pure Red Maasai but had better carcass grades. Red Maasai lambs had a higher dressing percentage and carcass fat level. Red Maasai lambs were undervalued by assessments of experienced evaluators by 8–13 % compared to pure Dorper based on empirical live weight and carcass weight payment schemes practised by the abattoir. Live weight was better than any other live measure in predicting carcass weight, which underpins the use of weighing scales when selecting lambs for slaughter and for breeding purposes. Based on the results of the crossbreds, Dorper might be useful as a terminal sire breed in crossing with Red Maasai ewes.
